# Validation of an automated sample preparation module directly connected to LC-MS/MS (CLAM-LC-MS/MS system) and comparison with conventional immunoassays for quantitation of tacrolimus and cyclosporin A in a clinical setting

**DOI:** 10.1186/s40780-023-00318-6

**Published:** 2024-01-08

**Authors:** Tsutomu Shimada, Daisuke Kawakami, Arimi Fujita, Rintaro Yamamoto, Satoshi Hara, Kiyoaki Ito, Ichiro Mizushima, Shinji Kitajima, Yasunori Iwata, Norihiko Sakai, Mitsuhiro Kawano, Takashi Wada, Yoshimichi Sai

**Affiliations:** 1 Department of Clinical Pharmacokinetics, Graduate School of Medical Sciences, Kanazawa University, Kanazawa, Ishikawa Japan; 2 Department of Hospital Pharmacy, University Hospital, Kanazawa University, 13-1 Takara-machi, Kanazawa, Ishikawa 920-8641 Japan; 3 Shimadzu Corporation, Kyoto, Japan; 4 Shimadzu Europa GmbH, Duisburg, Germany; 5 Department of Rheumatology, Graduate School of Medical Sciences, Kanazawa University, Kanazawa, Ishikawa Japan; 6 Department of Nephrology and Laboratory Medicine, Faculty of Medicine, Institute of Medical, Pharmaceutical and Health Sciences, Graduate School of Medical Sciences, Kanazawa University, Kanazawa, Ishikawa Japan; 7 AI Hospital/Macro Signal Dynamics Research and Development Center, Kanazawa University, Kanazawa, Ishikawa Japan

**Keywords:** CLAM-LC-MS/MS, Automatic pretreatment, Immunoassay, Tacrolimus, Cyclosporin A

## Abstract

**Background:**

Therapeutic drug monitoring (TDM) systems generally use either liquid chromatography/tandem mass spectrometry (LC-MS/MS) or immunoassay, though both methodologies have disadvantages. In this study, we aimed to evaluate whether a CLAM-LC-MS/MS system, which consists of a sample preparation module directly connected to LC-MS/MS, could be used for clinical TDM work for immunosuppressive drugs in whole blood, which requires a hemolytic process. For this purpose, we prospectively validated this system for clinical measurement of tacrolimus and cyclosporin A in patients’ whole blood. The results were also compared with those of commercial immunoassays.

**Methods:**

Whole blood from patients treated with tacrolimus or cyclosporin A at the Department of Nephrology and Departments of Rheumatology, Kanazawa University Hospital, from May 2018 to July 2019 was collected with informed consent, and drug concentrations were measured by CLAM-LC-MS/MS and by chemiluminescence immunoassay (CLIA) for tacrolimus and affinity column-mediated immunoassay (ACMIA) for cyclosporin A. Correlations between the CLAM-LC-MS/MS and immunoassay results were analyzed.

**Results:**

Two hundred and twenty-four blood samples from 80 patients were used for tacrolimus measurement, and 76 samples from 21 patients were used for cyclosporin A. Intra- and inter-assay precision values of quality controls were less than 7%. There were significant correlations between CLAM-LC-MS/MS and the immunoassays for tacrolimus and cyclosporin A (Spearman rank correlation coefficients: 0.861, 0.941, P < 0.00001 in each case). The drug concentrations measured by CLAM-LC-MS/MS were about 20% lower than those obtained using the immunoassays. CLAM-LC-MS/MS maintenance requirements did not interfere with clinical operations. Compared to manual pretreatment, automated pretreatment by CLAM showed lower inter-assay precision values and greatly reduced the pretreatment time.

**Conclusions:**

The results obtained by CLAM-LC-MS/MS were highly correlated with those of commercial immunoassay methods. CLAM-LC-MS/MS offers advantages in clinical TDM practice, including simple, automatic pretreatment, low maintenance requirement, and avoidance of interference.

**Supplementary Information:**

The online version contains supplementary material available at 10.1186/s40780-023-00318-6.

## Background

Therapeutic drug monitoring (TDM) in a clinical setting enables patient-specific dosing, and is critical for drugs such as immunosuppressants, antiepileptic drugs, and anticancer drugs, for which the therapeutic range is narrow, and which have serious, concentration-dependent side effects and large inter-individual variability. There are two main types of analytical methods for general TDM: immunoassay methods that utilize specific antibodies and separation-analysis methods such as liquid chromatography/tandem mass spectrometry (LC-MS/MS). The advantages of commercial immunoassay include rapidity and automation, as well as a high level of technical support from manufacturers [1]. Disadvantages include potential for cross-reactions, high cost per sample, inability to simultaneously measure multiple compounds, and inter-laboratory variability [2, 3]. However, in recent years, new immunoassay methods have ameliorated some of these problems [4, 5]. In contrast, LC-MS/MS is considered a worldwide gold standard method for TDM because of its high sensitivity, high specificity, and suitability for multiplexing including metabolites [6–8]. Nevertheless, it has disadvantages such as unsuitability for direct injection, limited automation, time-consuming procedure, the need for manual deproteination of samples, and the requirement of special training for users. To overcome some of these disadvantages, one-step protein precipitation methods such as Rapid-fire, liquid-handling platforms, on-line column extraction, and automated systems have been developed [9–13]. Nevertheless, to extend the availability of LC-MS/MS for clinical TDM, it is necessary to implement pretreatment automation, provide a user-friendly system, and ensure the availability of rapid technical support.

The CLAM-LC-MS/MS system, which is composed of an automated pretreatment device for blood (CLAM) directly connected to an LC-MS/MS instrument, has already been employed to measure drugs of abuse [14, 15], metabolite biomarkers [16], anti-HIV drugs [17], uracil and dihydrouracil [18], organic acids [19], and immunosuppressive drugs [20].

Immunosuppressive drugs bind extensively to red blood cells [21, 22], so a hemolysis process is required for clinical TDM work with whole blood samples. Therefore, in this work we aimed to establish whether CLAM-LC-MS/MS could be employed for this purpose by using it for the quantitation of tacrolimus and cyclosporin A in whole blood. We also compared the results with those of conventional immunoassays.

## Methods

### Clinical samples

Inpatients and outpatients who were treated with tacrolimus (Prograf Capsules or Graceptor Capsules; Astellas Pharma US) or cyclosporin A (Neoral; Novartis Pharma) at the Department of Nephrology and Department of Rheumatology, Kanazawa University Hospital, from May 2018 to July 2019 were recruited with informed consent. The present study was performed after receiving approval from the Medical Ethics Committee of Kanazawa University (protocol no. 2017 − 195).

### Equipment

The CLAM-LC-MS/MS system consists of a CLAM-2000 CL automated sample pretreatment device, CBM-20A CL system controller, CTO-20AC CL column oven, SIL-30AC CL autosampler, two LC-30AD CL pumps, FCV-20AH2 CL valve, DGU-20A5R CL degasser and LCMS-8050 CL (Shimadzu Corporation, Kyoto, Japan), all of which are approved for use as medical equipment. A DOSIMMUNE trap column (trap column) and a DOSIMMUNE analytical column (Alsachim, France) were used in the LC system. Commercial DOSIMMUNE kits (Alsachim, France) including calibrators (6 concentrations), 4 QC samples, and stable isotope-labeled internal standards (ISs; [^13^C_2_, ^2^H_4_]-everolimus, [^13^ C, ^2^H_3_]-sirolimus, [^13^ C, ^2^H_4_]-tacrolimus, and [^2^H_12_]-cyclosporin A) for everolimus, sirolimus, tacrolimus and cyclosporin A were used respectively. The validation and acceptable ranges for QC samples are shown in supplemental Table 1.

A chemiluminescence immunoassay (CLIA) method was applied to measure tacrolimus using the Architect system (ARCHITECT i1000SR, Abbott, Illinois, U.S.A) with an ARCHITECT Tacrolimus Reagent Kit, ARCHITECT Tacrolimus Calibrators, ARCHITECT Tacrolimus Whole Blood Precipitation Reagent, and Multichem WBT (Technopath Clinical Diagnostics, Ireland). An affinity column-mediated immune assay (ACMIA) method was applied to measure cyclosporin A, using Dimension® Xpand Plus (Siemens, Germany) with Dimension Systems CSA Assay, Dimension CSA Calibrator, and MORE RAP/Tac/CsA Control (More Diagnostics, CA. USA).

### Sample preparation

Whole blood collected from patients was divided into two tubes containing EDTA-Na. One was stored at -30˚C for CLAM-LC-MS/MS measurement and the other was stored at 4˚C for immunoassay. For application to the CLAM-LC-MS/MS system, the frozen whole blood samples (95 µL) were thawed at room temperature for hemolysis and placed in the CLAM unit. The CLAM unit was programmed to perform pretreatment, including sample extraction and protein precipitation followed by filtration and sample collection. First, 20 µL of 75% 2-propanol was dispensed onto the filter to activate the dedicated hydrophobic filter. Next, 20 µL of whole blood sample, 150 µL of extraction buffer, and 12.5 µL of ISs were dispensed into a dedicated vial and stirred for 60 s. The samples were vacuum-filtered (approximately 50 to 60 kPa) for 60 s in a dedicated filter consisting of a polytetrafluoroethylene membrane with a pore size of 0.45 μm and collected in a collection vial. Finally, the filtrate was automatically transported to the HPLC unit for LC-MS/MS analysis. The MS/MS conditions are shown in Table 1.

**Table 1:** Optimised mass spectrometry parameters of tacrolimus, cyclosporin A, and ISs in CLAM-LC-MS/MS

	Precursor ion				Product ion					
Compounds			Quantitation				Reference			Retention time (min)
	m/z		Q1	Collision	Q3		Q1	Collision	Q3	
		***m/z***	**pre-bias**	**energy**	**pre-bias**	***m/z***	**pre-bias**	**energy**	**pre-bias**	
			**(V)**	**(V)**	**(V)**		**(V)**	**(V)**	**(V)**	
Tacrolimus	821.5	768.3	-30	-22	-22	576.2	-30	-22	-30	0.735
[^13^ C,^2^H_4_]-Tacrolimus	826.5	773.4	-30	-22	-22	581.4	-30	-22	-30	0.735
Cyclosporin A	1219.8	1202.9	-36	-19	-32	1184.6	-38	-35	-36	0.783
[^2^H_12_]-Cyclosporin A	1231.7	1214.9	-36	-19	-32	1196.8	-38	-35	-36	0.780

For measuring tacrolimus with CLIA, sample preparation was conducted according to the manufacturer’s protocol. Briefly, Whole Blood Precipitation Reagent was added into the same volume (200 µL) of whole blood samples, QCs, or calibrators. After vortexing and centrifugation, the supernatant was immediately transferred to the Architect system. To measure cyclosporine A with ACMIA, whole blood samples (250 µL) were directly placed in the carousel of the Dimension system.

### Maintenance

Regular maintenance of the CLAM-LC-MS/MS system was performed every 6 months as follows. The flow path of LC, lens system of the mass spectrometer, and sample probe of CLAM were cleaned, and the capillary, desolvation line, and PEEK tube were replaced. Technical support was promptly available if required at other times.

### Statistical analysis

Statistical analyses were performed with MedCalc statistical software for Windows (Ostend, Belgium). Data collected by different assay methods were compared by means of Passing and Bablok regression analysis [23] and calculation of Spearman correlation coefficients. The Bland-Altman approach was used to compare immunological assay and CLAM-LC-MS/MS assay results by plotting the relative differences [24].　A p-value of 0.05 was considered statistically significant.

## Results

### Blood samples

Between May 2018 and July 2019, 101 Japanese patients (18 in the Department of Nephrology and 83 in the Department of Rheumatology at Kanazawa University Hospital) gave informed consent to participate in this study. Finally, 224 whole blood samples were obtained from 80 patients treated with tacrolimus; 73 with Prograf (Astellas Pharma, Japan), and 7 with Graceptor (Astellas Pharma, Japan). Among these patients, 18 were inpatients and 62 were outpatients. In addition, 76 whole blood samples were obtained from 21 patients treated with cyclosporin A (8 were inpatients, 13 were outpatients), all of whom received Neoral (Novartis Japan). All whole blood samples were collected at times corresponding to a trough concentration.

### CLAM-LC-MS/MS assay

The analysis time from placing the sample in the CLAM unit to obtaining the result with CLAM-LC-MS/MS was about 10 min; 6 min for pretreatment by CLAM, 2.6 min for LC-MS/MS analysis, and 30 s for result output. In the case of multiple continuous measurements, since pretreatment of each subsequent sample with CLAM was performed during the LC-MS/MS analysis of the previous sample, a measurement could be performed about every 4 min from the second sample on. For example, the measurement of a total of 40 samples including 20 actual samples, 6 calibration standards and 4 QCs (measured twice, before and after sample measurement) took approximately 180 min, while CLIA took about 60 min and ACMIA took about 40 min to measure the same sample number. Pretreatment of whole blood manually instead with CLAM in the LC-MS/MS method took 90 min longer (20 samples). The intra- and inter-assay precision values of four QCs of tacrolimus and cyclosporin A were less than 7% (Table 2). The values of carry-over of LLOQ (lower limit of quantitation) were 4.4% for tacrolimus and 9.6% for cyclosporin A. The measured concentrations of QCs in DOSIMMUNE kits of tacrolimus measured by the CLIA method were within the acceptable range (± 20%). The inter-assay precision values of QCs of tacrolimus in the case of manual pretreatment methods were higher (QC1 15.3%, QC2 13.4, QC3 15.4%, QC4 17.2%) than those obtained in the case of automated pretreatment by CLAM (Table 2).

**Table 2:** Results of the validation study of QCs (tacrolimus and cyclosporin A) in CLAM-LC-MS/MS

Compounds	Calibration curve Dynamic range (ng/mL)	r^2^	LLOQ (ng/mL)	Intra-assay precision (%) (%RSD) (n = 10)				Inter-assay precision (%) (%RSD) (10 days)			
				C1	C2	C3	C4	C1	C2	C3	C4
Tacrolimus	2.0-35.6	0.999	2.0	97.3 (6.5)	100.4 (3.5)	100.4 (2.6)	105.5 (3.6)	94.8 (3.1)	99.8 (4.7)	101.7 (4.4)	105.9 (5.1)
Cyclosporin A	24.3-1838.6	0.999	24.3	95.3 (2.5)	97.7 (1.6)	97.7 (1.9)	104.3 (1.6)	98.8 (4.0)	99.0 (3.7)	96.6 (3.6)	98.7 (4.5)

LLOQ: low limit of quantification, RSD: relative standard deviation

### Correlation between CLAM-LC-MS/MS and immunoassay results

**Fig. 1:**
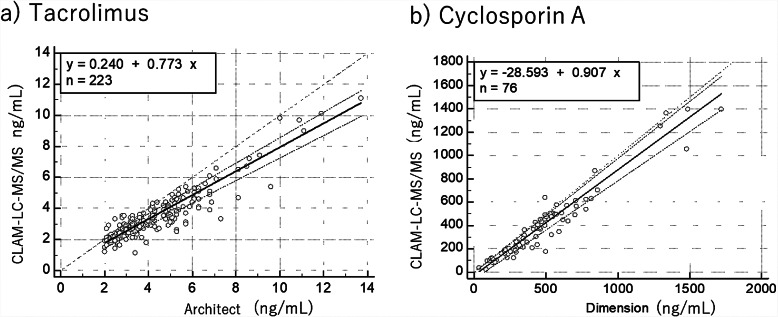
Passing-Bablok correlations between the CLAM-LC-MS/MS and immunoassays. **a**) Tacrolimus (n = 223). **b**) Cyclosporin A (n = 76). The regression lines are shown as solid lines, and the 95% confidence limits are shown as dashed lines. The dotted lines are the lines of identity

**Fig. 2:**
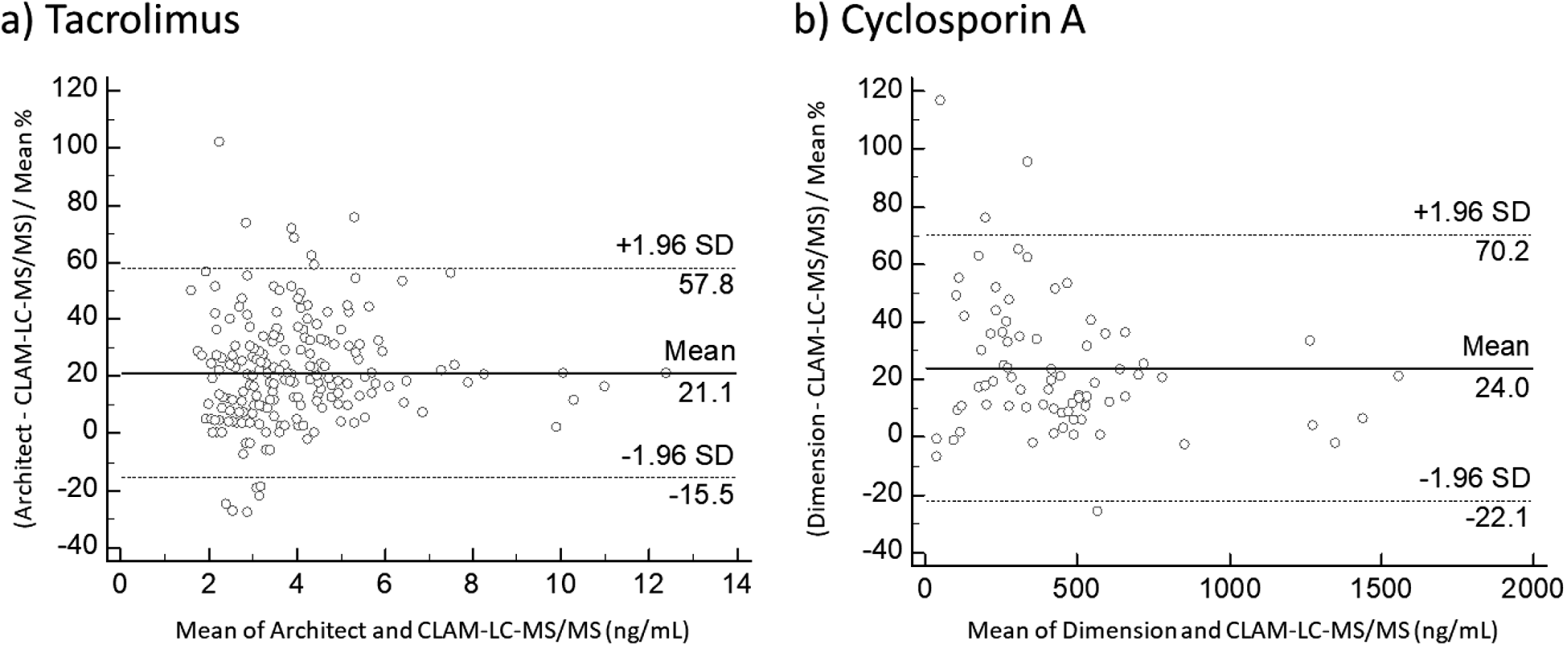
Bland-Altman plots to identify relative differences between CLAM-LC-MS/MS and immunoassays. **a**) Tacrolimus (n = 223). **b**) Cyclosporin A (n = 76). Mean differences are shown as solid lines and 95% confidence limits are shown as dashed lines

The correlations between CLAM-LC-MS/MS and immunoassay results for tacrolimus and cyclosporin A were analyzed in terms of the Passing-Bablok correlation and Bland-Altman plots (Figs. 1 and 2). A significant correlation was obtained between CLAM-LC-MS/MS and CLIA for tacrolimus (Spearman rank correlation coefficient: 0.861, P < 0.00001). The Passing-Bablok intercept of the linear regression was 0.2398 (95% CI: 0.01818 to 0.3828), and the slope was 0.773 (95% CI: 0.7241 to 0.8182). Constant error and proportional error were observed, and the concentration data obtained by CLAM-LC-MS/MS were 21.1% (from − 15.5 to 57.8%) lower than those measured by CLIA. Large variations were observed, especially in the low concentration range.

A significant correlation was also obtained between CLAM-LC-MS/MS and ACMIA for cyclosporin A (Spearman rank correlation coefficient: 0.941, P < 0.00001). The Passing-Bablok intercept of the linear regression was − 28.5933 (95% CI: -58.5836 to -6.6225), and the slope was 0.9066 (95% CI: 0.8425 to 0.9786). Constant error and proportional error were observed, and the concentration data obtained by CLAM-LC-MS/MS were 24% (from − 22.1 to 70.2%) lower than those obtained by ACMIA. Large variations were observed, especially in the low concentration range.

### Maintenance

Regular maintenance was performed every 6 months and completed within 2 days. Incidental problems, such as clogging of the sampling probe or position adjustment error of the sampling probe, that occurred outside scheduled maintenance were dealt with by technical support within one day.

## Discussion

Measurement of the whole blood concentration of immunosuppressive drugs in clinical samples requires hemolysis and pretreatment processes. Our results in this study show that CLAM-LC-MS/MS offers advantages in clinical TDM practice, including simple, automatic pretreatment, low maintenance requirements, and avoidance of interference. We also confirmed that the values measured by CLAM-LC-MS/MS were highly correlated with those obtained by immunological assays.

Compared to manual pretreatment of whole blood samples, automated pretreatment by CLAM showed lower inter-assay precision values of the QCs and greatly reduced the pretreatment time. Thus, automated pretreatment by CLAM should be advantageous in the application of LC-MS/MS for clinical TDM. The CLAM-LC-MS/MS system requires hemolysis of blood samples by pre-freezing and thawing, but once the sample is placed in the CLAM unit, all subsequent processing is automatically performed, eliminating variability due to differences in the operator’s experience. The CLIA method for measurement of tacrolimus requires a pretreatment process that includes the addition of deproteinizing agent, mixing, and centrifugation. On the other hand, the ACMIA method enables measurement of cyclosporin A concentration in whole blood, so that samples can be directly placed in the carousel. However, ACMIA has disadvantages, such as high measurement error and low minimum detection sensitivity (about 5 times higher than CLIA), suggesting that the elimination of hemolysis and deproteinization processes may not be appropriate for TDM [2, 25]. In this study, we conducted hemolysis by freezing the whole blood samples at -30 °C overnight and thawing them at room temperature, but this might not be suitable for clinical samples, where rapid feedback to clinical staff is important for prompt dosing design. Recently, a hemolysis method for whole blood that involves freezing at -80 °C for just 10 min and thawing in running water for 5 min was reported and was employed to measure tacrolimus by CLAM-LC-MS/MS [20]. Furthermore, since there are several hemolysis methods [26], it will be necessary to optimize and validate the hemolysis method for the measurement of tacrolimus and cyclosporin A by CLAM-LC-MS/MS in clinical practice.

We found that the blood levels of tacrolimus and cyclosporin A in clinical samples measured by CLAM-LC-MS/MS and immunoassay were significantly correlated. However, the Bland-Altman plot for both tacrolimus and cyclosporin A indicated that the concentrations measured by CLAM-LC-MS/MS were 20% lower than those by the immunoassays. It is well known that immunoassays often show cross-reactivity with metabolites of parent compounds and the extent of the cross-reactivity is variable, depending on the antibodies used [27]. In previous studies, LC-MS/MS also showed a 10–20% lower value than immunoassay [28, 29], in agreement with the present finding. This difference should be noted when updating from immunoassay to LC-MS/MS in a clinical setting, or when a patient moves to another facility where a different method is in use.

Appropriate maintenance is also a critical point for TDM, since continuous availability of analysis is clinically important. In this study using whole blood samples, increased column pressure and clogging in the sampling probe each occurred once, but did not occur after modifying the washing process to include alkali detergent, indicating that this modification successfully enabled the use of CLAM-LC-MS/MS for whole blood samples. The combination of regular maintenance and prompt technical support to deal with incidental problems was effective to maintain availability of the instrument.

Furthermore, the commercial CLAM-LC-MS/MS system is provided with standards, QCs, and stable isotope ISs, but it remains necessary to conduct multi-facility studies to confirm consistency across multiple facilities in the future.

## Conclusions

CLAM-LC-MS/MS showed high robustness for the measurement of tacrolimus and cyclosporin A in whole blood samples, which require hemolysis and pretreatment, and the results were highly correlated with those of conventional immunoassay systems. Importantly, the commercial CLAM-LC-MS/MS system, which includes standards, QCs, ISs, and technical support, is user-friendly, and pretreatment is automatic, so that the required technical expertise is minimal. Our results suggest that CLAM-LC-MS/MS would be suitable for routine TDM operation, and would reduce the burden on clinical pharmacists, though further multi-facility validation remains necessary.

### Electronic supplementary material

Below is the link to the electronic supplementary material.

Supplementary Material 1

## Data Availability

Not applicable.

## List of abbreviations

LC-MS/MS: Liquid chromatography/tandem mass spectrometry
CLAM: Automated pretreatment device for blood
TDM: Therapeutic drug monitoring
QC: Quality control
IS: Internal standard
CLIA: Chemiluminescence immunoassay
ACMIA: Affinity column-mediated immune assay
LLOQ: Low limit of quantitation
LOD: Limit of detection
RSD: Relative standard deviation
