# Supplementary material for: Validation of an automated sample preparation module directly connected to LC-MS/MS (CLAM-LC-MS/MS system) and comparison with conventional immunoassays for quantitation of tacrolimus and cyclosporin A in a clinical setting
Source: J Pharm Health Care Sci. 2024 Jan 8;10:5. doi: 10.1186/s40780-023-00318-6 (PMC10773076; doi:10.1186/s40780-023-00318-6)
Supplement: Supplementary file 1 — Supplementary Material 1 [file 40780_2023_318_MOESM1_ESM.docx]

Supplemental Table 1 QC validation studies in CLAM-LCMSMS and acceptable ranges for QC samples.

| Compound |  | Intra-assay (ng/mL) | | | | Inter-assay (ng/mL) | | | |
| --- | --- | --- | --- | --- | --- | --- | --- | --- | --- |
|  |  | C1 | C2 | C3 | C4 | C1 | C2 | C3 | C4 |
| Tacrolimus | Set conc. | 3.60 | 8.10 | 12.4 | 22.5 | 4.20 | 8.80 | 12.6 | 19.2 |
|  | Ave. conc. | 3.50 | 8.83 | 12.8 | 20.3 | 3.98 | 8.78 | 12.8 | 20.3 |
|  | QC Range  (±15%) | 3.10-  4.10 | 7.50-  10.1 | 10.7-  14.5 | 16.3-  22.1 | 3.57-  4.83 | 7.48-  10.1 | 10.7-  14.5 | 16.3-  22.1 |

| Compound |  | Intra-assay (ng/mL) | | | | Inter-assay (ng/mL) | | | |
| --- | --- | --- | --- | --- | --- | --- | --- | --- | --- |
|  |  | C1 | C2 | C3 | C4 | C1 | C2 | C3 | C4 |
| Cyclosporin A | Set conc. | 42.3 | 166.7 | 822.9 | 1372 | 47.8 | 166.7 | 822.9 | 1372 |
|  | Ave. conc. | 40.3 | 162.8 | 825.8 | 1430 | 47.2 | 165.1 | 794.5 | 1353 |
|  | QC Range  (±15%) | 36.0-  48.6 | 141.7-  191.7 | 699.5-  946.3 | 1166-  1577 | 40.6-  55.0 | 141.7-  191.7 | 699.5-  946.3 | 1166.2-  1577.8 |
